# (*Z*)-2,2,2-Trichloro-*N* ^2^-cyano­acetamidine

**DOI:** 10.1107/S1600536809031079

**Published:** 2009-08-12

**Authors:** A. Elizabeth Baker, René T. Boeré

**Affiliations:** a Department of Chemistry and Biochemistry, The University of Lethbridge, Lethbridge, AB, Canada T1K 3M4

## Abstract

The title compound, C_3_H_2_Cl_3_N_3_, crystallizes as the *Z* isomer with respect to the C=N bond. The –C(NH_2_)=NCN functional group is effectively planar (r.m.s. deviation = 0.016 Å), with only the three Cl atoms out of the mol­ecular plane. A strong network of N—H⋯N hydrogen bonds forms dimers which are associated into ribbons in the crystal structure. Hydrogen bonding is suspected to be the cause of the near-equivalence of the formal C—N and C=N bonds (Δ_CN_ = 0.008 Å)

## Related literature

For literature related to characterization, see: Huffman & Schaefer (1963 ▶). For comparable structures of *N*′-cyano­amidines; see Allen (2002 ▶). For the crystal structures of *N* ^2^-cyano-3-[2-diamino­methyl­eneamino)-4-thia­zolylmethyl­thio]propionamidine­monohydrate, (II) and 3-{2-[amino­(methyl­amino)methyl­eneamino]-4-thia­zolylmethyl­thio}-*N* ^2^-cyano­propionamidine, (III), see Ishida *et al.* (1989 ▶). For the crystal structure of (*E*)-1,2-bis­(1-amino-1-(cyano­imino)-2-methyl­prop-2-yl)diazene-1,2- dioxide, (IV), see: Tretyakov *et al.* (2006 ▶). For the sole other acyclic trichloro­methyl amidine with a reported crystal structure, *N*-(4-amino-3-furan­zanyl)-2,2,2-trichloro-*N*-methoxy­acetamidine, (V), see: George & Gilardi (1986 ▶). For background to the Δ_CN_ parameter, see: Boeré, *et al.* (1998 ▶).
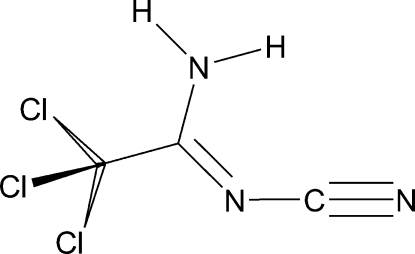


## Experimental

### 

#### Crystal data

C_3_H_2_Cl_3_N_3_
*M* *_r_* = 186.43
Monoclinic, 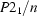

*a* = 5.5388 (4) Å
*b* = 6.6127 (4) Å
*c* = 18.4727 (12) Å
β = 95.122 (1)°
*V* = 673.89 (8) Å^3^
*Z* = 4
Mo *K*α radiation
μ = 1.26 mm^−1^
*T* = 173 K
0.41 × 0.27 × 0.21 mm

#### Data collection

Bruker APEXII CCD area-detector diffractometer
Absorption correction: multi-scan (*SADABS*; Bruker, 2006 ▶) *T*_min_ = 0.616, *T*_max_ = 0.770
7459 measured reflections
1552 independent reflections
1479 reflections with *I* > 2σ(*I*)
*R*_int_ = 0.017

#### Refinement

*R*[*F* ^2^ > 2σ(*F* ^2^)] = 0.019
*wR*(*F* ^2^) = 0.050
*S* = 1.06
1552 reflections
83 parameters
H-atom parameters constrained
Δρ_max_ = 0.41 e Å^−3^
Δρ_min_ = −0.27 e Å^−3^

### 

Data collection: *APEX2* (Bruker, 2006 ▶); cell refinement: *SAINT-Plus* (Bruker, 2006 ▶); data reduction: *SAINT-Plus*; program(s) used to solve structure: *SHELXS97* (Sheldrick, 2008 ▶); program(s) used to refine structure: *SHELXTL* (Sheldrick, 2008 ▶); molecular graphics: *Mercury* (Macrae *et al.*, 2006 ▶); software used to prepare material for publication: *publCIF* (Westrip, 2009 ▶).

## Supplementary Material

Crystal structure: contains datablocks I, global. DOI: 10.1107/S1600536809031079/wn2341sup1.cif

Structure factors: contains datablocks I. DOI: 10.1107/S1600536809031079/wn2341Isup2.hkl

Additional supplementary materials:  crystallographic information; 3D view; checkCIF report

## Figures and Tables

**Table 1:** Hydrogen-bond geometry (Å, °)

*D*—H⋯*A*	*D*—H	H⋯*A*	*D*⋯*A*	*D*—H⋯*A*
N1—H1*A*⋯N3^i^	0.88	2.10	2.9583 (15)	164
N1—H1*B*⋯N3^ii^	0.88	2.40	3.1893 (15)	150

Symmetry codes: (i) 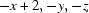; (ii) 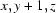.

**Table 2:** Comparative distances (Å) and angles (°) in amidines (I)–(V)

Value	(I)	(II)	(III)	(IV)	(V)	
C2—N1	1.3115 (15)	1.308 (4)	1.308 (3)	1.307 (2)	1.387 (4)	
C2—N2	1.3032 (15)	1.320 (4)	1.317 (3)	1.306 (2)	1.2737 (4)	
Δ_CN_	0.008	−0.012	−0.009	0.001	0.114	
C2—C1	1.5396 (15)	1.520 (4)	1.513 (3)	1.522 (3)	1.525 (5)	
C3—N3	1.1533 (17)	1.164 (4)	1.1567 (3)	1.153 (3)		
C3—N2	1.3226 (16)	1.320 (4)	1.333 (3)	1.322 (3)		
N2—C2—N1	127.94 (11)	118.0 (2)	125.9 (2)	126.0 (2)	127.7 (3)	
N2—C2—C1	114.43 (10)	124.1 (2)	116.8 (2)	114.9 (1)	117.2 (3)	
N1—C2—C1	117.52 (10)	117.9 (2)	117.3 (2)	118.7 (1)	115.0 (3)	
C2—N2—C3	121.04 (10)	119.1 (2)	118.7 (1)			
N3—C3—N2	172.16 (13)	173.2 (2)	173.9 (3)	173.2 (2)		

**Fig. 1.**
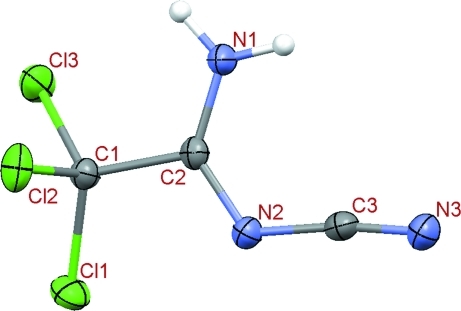
A view of (I), plotted with displacement ellipsoids drawn at the 50% probability level. H atoms are shown as spheres of arbitrary radius.

Compound (I) corresponds to the title compound and (II)–(V) are defined in the *Related Literature* section. Atom numbering corresponds to that in Fig. 1 (in Supplementary materials).

**Fig. 2.**
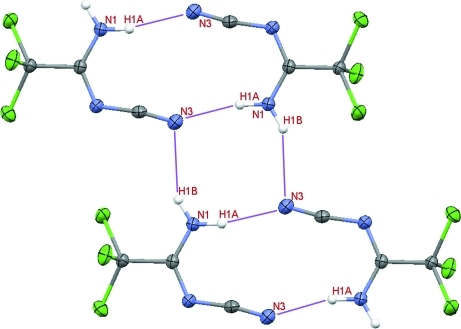
The network of hydrogen bonds (dashed lines) linking centrosymmetric pairs of molecules into planar ribbons along the b axis. Symmetry equivalents are -x, 1 + y, -z; 2 - x, -y, -x and 2 - x, 1 - y, -x. These ribbons lie parallel to the (207) Miller planes.

## supplementary crystallographic information

### Comment

The stucture of the title compound, (I), is shown in Fig. 1. Molecular dimensions are available in the archived CIF. Structure (I) crystallizes as the *Z* isomer with respect to the imino bond (Fig. 1). The structure is essentially planar except for the CCl_3_ group (r.m.s. mean deviation for the —C(NH_2_)═NCN group is 0.016 Å), while Cl2 is almost perpendicular to this plane; thus Cl1 deviates by 0.65 and Cl3 by 0.84 Å from the plane. The parameter Δ_CN_ = *d*(C—N) - *d*(C═N) has been found to range between 0 and 0.178 Å for many amidines for which the structures are known (Boeré *et al.*, 1998). For (I), Δ_CN_ = 0.008 Å, which is very small for a monomeric amidine with such unsymmetrical substitution. The N2–C3–N3 angle is almost linear, at 172.16 (13)°. There is a network of N—H···N hydrogen bonds (Table 1) linking centrosymmetric pairs of molecules into planar ribbons along the *b* axis (Fig. 2). Short contacts of 3.203 (1) Å between Cl2 (the upward- and downward-facing chlorine atoms) and N2 (imino nitrogen) link these layers into a 3-D network in the crystal structure. Finally, there are 3.4132 (5) Å short contacts between Cl1 and Cl2 bridging molecules. It is likely that this strong intermolecular hydrogen bonding is responsible for the small value of Δ_CN_.

Of nine *N*'-cyanoamidines in the literature, six are *E* (refcodes HANBAA, ILIPAU, JATLIZ, TAHHOA, TESQAK, WAXXUO; Allen, 2002) and two are *Z* (refcodes JATMAS, NERKAX; Allen, 2002) with respect to the imino bond; for the last, VOVPUR (Allen, 2002), the isomer is not specified. The most relevant for comparison with (I) are *N*^2^-cyano-3-[2-diaminomethyleneamino)-4-thiazolylmethylthio]propionamidinemonohydrate, (II), 3-{2-[amino(methylamino)methyleneamino]-4-thiazolylmethylthio}-*N*^2^-cyanopropionamidine, (III) (Ishida *et al.*, 1989) and (*E*)-1,2-bis(1-amino-1-(cyanoimino)-2-methylprop-2-yl)diazene-1,2- dioxide, (IV) (Tretyakov *et al.*, 2006), which all bear the *N*H_2_ group in addition to the nitrile on *N*'. Each of these structures shares the high degree of planarity of the –C(NH_2_)═NCN group (r.m.s. deviations for (II) - (IV) are 0.008, 0.025 and 0.069 Å, respectively.) Of these three examples, (II) is *E* while (III) and (IV) are both *Z*; note that (II) and (III) differ only in methylation at a very remote amino group. There is only one acyclic trichloromethyl amidine with a crystal structure reported in the literature, viz. *N*-(4-amino-3-furanzanyl)-2,2,2-trichloro-*N*-methoxyacetamidine, (V) (George & Gilardi, 1986) and this is the *Z *isomer. The structure of (IV), which is arguably the most similar structure, electronically and chemically, to (I) also shows a very similar pattern of hydrogen bonding where centrosymetric dimers are linked in ribbons within the crystal structure by additional hydrogen bonds.

Key geometrical parameters for structures (I) - (V) are compared in Table 2, which includes values for Δ_CN_, all of which fall within the known range. However, (II) and (III) are highly unusual in having the wrong sign for this parameter. That is, the imino bond is actually longer than the amino. We are not aware of other instances of this occurrence; the locations of the NH_2_ hydrogen atoms in both structures were corroborated by expected hydrogen bonding. It is likely that this powerful hydrogen bonding is responsible for the inversion in expected bond distances, perhaps augmented by the strong electron-withdrawing cyano subsituent on N'.

### Experimental

General Procedures: Reagent grade methanol was dried by distillation with Mg and catalytic I_2_. Sodium methoxide was transferred to the flask within a glove box under nitrogen.

Preparation of methyl trichloroacetimidate: 50 ml of dried methanol and 21.66 g (150 mmol) of trichloroacetonitrile were added to 0.50 g (10 mmol) of sodium methoxide. After stirring for 48 h at room temperature, the solution was saturated with CO_2_(*s*) to eliminate remaining sodium methoxide. Methanol was then distilled off at 335–7 K, whereafter the liquid methyl trichloroacetimidate was distilled at 415 K at a reduced pressure. Yield 19.59 g (110 mmol, 74%).

Preparation of 2,2,2-trichloro-*N*'-cyano-acetamidine: 0.42 g (10 mmol) of cyanamide was dissolved in 5 ml of anhydrous methanol. With stirring, 1.76 g (10 mmol) of methyl trichloroacetimidate was added dropwise. An ice bath may be required to maintain temperature during addition of methyl trichloroacetimidate. The solution was stirred for 3 h at RT. Methanol was removed by rotary evaporation followed by high vacuum. The solid residue was dissolved in a minimum volume (3.5 ml) of hot CH_3_CN, cooled to room temperature, and placed within the 238 K freezer. The colourless crystals produced were filtered and vacuum dried yielding 0.121 g (0.649 mmol, 6.51% yield), mp 433–7 K (Huffman & Schaefer, 1963).

### Refinement

Both H atoms were located in a difference Fourier map. They were refined using a riding model and U_iso_(H) was set equal to 1.2U_eq_(N1). The highest residual peak has a fraction of the electron density of a single H atom and is located 0.76 Å from Cl1.

### Crystal data

**Table:**

C_3_H_2_Cl_3_N_3_	*F*(000) = 368
*M**_r_* = 186.43	*D*_x_ = 1.838 Mg m^−^^3^
Monoclinic, *P*2_1_/*n*	Melting point: 441 K
Hall symbol: -P 2yn	Mo *K*α radiation, λ = 0.71073 Å
*a* = 5.5388 (4) Å	Cell parameters from 4497 reflections
*b* = 6.6127 (4) Å	θ = 2.2–27.6°
*c* = 18.4727 (12) Å	µ = 1.26 mm^−^^1^
β = 95.122 (1)°	*T* = 173 K
*V* = 673.89 (8) Å^3^	Block, colourless
*Z* = 4	0.41 × 0.27 × 0.21 mm

### Data collection

**Table:**

Bruker APEXII CCD area-detector diffractometer	1552 independent reflections
Radiation source: Molybdenum	1479 reflections with *I* > 2σ(*I*)
graphite	*R*_int_ = 0.017
φ and ω scans	θ_max_ = 27.5°, θ_min_ = 2.2°
Absorption correction: multi-scan (*SADABS*; Bruker, 2006)	*h* = −7→7
*T*_min_ = 0.616, *T*_max_ = 0.770	*k* = −8→8
7459 measured reflections	*l* = −24→23

### Refinement

**Table:**

Refinement on *F*^2^	Secondary atom site location: difference Fourier map
Least-squares matrix: full	Hydrogen site location: difference Fourier map
*R*[*F*^2^ > 2σ(*F*^2^)] = 0.019	H-atom parameters constrained
*wR*(*F*^2^) = 0.050	*w* = 1/[σ^2^(*F*_o_^2^) + (0.0247*P*)^2^ + 0.2765*P*] where *P* = (*F*_o_^2^ + 2*F*_c_^2^)/3
*S* = 1.06	(Δ/σ)_max_ = 0.001
1552 reflections	Δρ_max_ = 0.41 e Å^−^^3^
83 parameters	Δρ_min_ = −0.27 e Å^−^^3^
0 restraints	Extinction correction: *SHELXTL* (Sheldrick, 2008), Fc^*^=kFc[1+0.001xFc^2^λ^3^/sin(2θ)]^-1/4^
Primary atom site location: structure-invariant direct methods	Extinction coefficient: 0.0231 (18)

### Special details

**Table:**

Geometry. All e.s.d.'s (except the e.s.d. in the dihedral angle between two l.s. planes) are estimated using the full covariance matrix. The cell e.s.d.'s are taken into account individually in the estimation of e.s.d.'s in distances, angles and torsion angles; correlations between e.s.d.'s in cell parameters are only used when they are defined by crystal symmetry. An approximate (isotropic) treatment of cell e.s.d.'s is used for estimating e.s.d.'s involving l.s. planes.
Refinement. Refinement of *F*^2^ against ALL reflections. The weighted *R*-factor *wR* and goodness of fit *S* are based on *F*^2^, conventional *R*-factors *R* are based on *F*, with *F* set to zero for negative *F*^2^. The threshold expression of *F*^2^ > σ(*F*^2^) is used only for calculating *R*-factors(gt) *etc*. and is not relevant to the choice of reflections for refinement. *R*-factors based on *F*^2^ are statistically about twice as large as those based on *F*, and *R*- factors based on ALL data will be even larger.

### Fractional atomic coordinates and isotropic or equivalent isotropic displacement parameters (Å^2^)

**Table:**

	*x*	*y*	*z*	*U*_iso_*/*U*_eq_	
Cl1	0.23790 (5)	0.15285 (5)	0.181208 (18)	0.02983 (10)	
Cl2	0.66794 (6)	0.37010 (5)	0.233709 (17)	0.02976 (10)	
Cl3	0.33903 (6)	0.53919 (5)	0.119806 (17)	0.02989 (10)	
C2	0.62444 (19)	0.20753 (17)	0.10178 (6)	0.0179 (2)	
C3	0.7632 (2)	−0.09854 (17)	0.06561 (7)	0.0224 (2)	
C1	0.4692 (2)	0.31232 (17)	0.15584 (6)	0.0196 (2)	
N2	0.62859 (18)	0.01089 (15)	0.10646 (5)	0.0229 (2)	
N1	0.74493 (18)	0.32334 (15)	0.05999 (6)	0.0230 (2)	
H1A	0.8430	0.2689	0.0305	0.028*	
H1B	0.7277	0.4555	0.0614	0.028*	
N3	0.8680 (2)	−0.21297 (17)	0.03320 (6)	0.0298 (2)	

### Atomic displacement parameters (Å^2^)

**Table:**

	*U*^11^	*U*^22^	*U*^33^	*U*^12^	*U*^13^	*U*^23^
Cl1	0.02508 (16)	0.02924 (17)	0.03733 (18)	−0.00196 (11)	0.01472 (13)	0.00338 (12)
Cl2	0.03001 (17)	0.03173 (17)	0.02701 (16)	0.00592 (12)	−0.00034 (12)	−0.01080 (12)
Cl3	0.03524 (18)	0.02289 (16)	0.03307 (17)	0.01263 (12)	0.01157 (13)	0.00600 (11)
C2	0.0173 (5)	0.0184 (5)	0.0181 (5)	0.0013 (4)	0.0022 (4)	−0.0011 (4)
C3	0.0260 (6)	0.0158 (5)	0.0259 (6)	−0.0021 (4)	0.0060 (5)	0.0019 (4)
C1	0.0199 (5)	0.0175 (5)	0.0221 (5)	0.0025 (4)	0.0054 (4)	0.0012 (4)
N2	0.0269 (5)	0.0166 (5)	0.0264 (5)	0.0015 (4)	0.0096 (4)	0.0000 (4)
N1	0.0270 (5)	0.0168 (5)	0.0270 (5)	0.0010 (4)	0.0121 (4)	0.0001 (4)
N3	0.0364 (6)	0.0194 (5)	0.0355 (6)	0.0012 (4)	0.0136 (5)	−0.0025 (4)

### Geometric parameters (Å, °)

**Table:**

Cl1—C1	1.7549 (12)	C2—C1	1.5396 (15)
Cl2—C1	1.7733 (12)	C3—N3	1.1533 (17)
Cl3—C1	1.7686 (12)	C3—N2	1.3226 (16)
C2—N2	1.3032 (15)	N1—H1A	0.8800
C2—N1	1.3115 (15)	N1—H1B	0.8800
			
N2—C2—N1	127.94 (11)	C2—C1—Cl2	106.31 (7)
N2—C2—C1	114.43 (10)	Cl1—C1—Cl2	109.15 (6)
N1—C2—C1	117.52 (10)	Cl3—C1—Cl2	108.98 (6)
N3—C3—N2	172.16 (13)	C2—N2—C3	121.04 (10)
C2—C1—Cl1	111.47 (8)	C2—N1—H1A	120.0
C2—C1—Cl3	111.73 (8)	C2—N1—H1B	120.0
Cl1—C1—Cl3	109.12 (6)	H1A—N1—H1B	120.0
			
N2—C2—C1—Cl1	−26.28 (12)	N2—C2—C1—Cl2	92.56 (10)
N1—C2—C1—Cl1	157.21 (9)	N1—C2—C1—Cl2	−83.95 (11)
N2—C2—C1—Cl3	−148.66 (9)	N1—C2—N2—C3	−0.9 (2)
N1—C2—C1—Cl3	34.83 (12)	C1—C2—N2—C3	−176.95 (10)

### Hydrogen-bond geometry (Å, °)

**Table:**

*D*—H···*A*	*D*—H	H···*A*	*D*···*A*	*D*—H···*A*
N1—H1A···N3^i^	0.88	2.10	2.9583 (15)	164
N1—H1B···N3^ii^	0.88	2.40	3.1893 (15)	150

Symmetry codes: (i) −*x*+2, −*y*, −*z*; (ii) *x*, *y*+1, *z*.

### Table 2 Comparative distances (Å) and angles (°) in amidines (I)–(V)

**Table:**

Value	(I)	(II)	(III)	(IV)	(V)	
C2-N1	1.3115 (15)	1.308 (4)	1.308 (3)	1.307 (2)	1.387 (4)	
C2-N2	1.3032 (15)	1.320 (4)	1.317 (3)	1.306 (2)	1.2737 (4)	
Δ_CN_	0.008	-0.012	-0.009	0.001	0.114	
C2-C1	1.5396 (15)	1.520 (4)	1.513 (3)	1.522 (3)	1.525 (5)	
C3-N3	1.1533 (17)	1.164 (4)	1.1567 (3)	1.153 (3)		
C3-N2	1.3226 (16)	1.320 (4)	1.333 (3)	1.322 (3)		
N2-C2-N1	127.94 (11)	118.0 (2)	125.9 (2)	126.0 (2)	127.7 (3)	
N2-C2-C1	114.43 (10)	124.1 (2)	116.8 (2)	114.9 (1)	117.2 (3)	
N1-C2-C1	117.52 (10)	117.9 (2)	117.3 (2)	118.7 (1)	115.0 (3)	
C2-N2-C3	121.04 (10)	119.1 (2)	118.7 (1)			
N3-C3-N2	172.16 (13)	173.2 (2)	173.9 (3)	173.2 (2)		

Atom numbering corresponds to that in Fig. 1.
